# Integrated mass drug administration for yaws eradication: evidence from a comparative observational study in Papua New Guinea and a systematic review with network meta-analysis

**DOI:** 10.1136/bmjgh-2026-023743

**Published:** 2026-05-13

**Authors:** Jingyuan Xu, Mary Yohogu, Francisca S Y Wong, Sarosh Jamil, Fukushi Morishita, Kazim Hizbullah Sanikullah, Rajendra Prasad Hubraj Yadav, Huong Thi Giang Tran

**Affiliations:** 1 World Health Organization Regional Office for the Western Pacific, Manila, Philippines; 2 Government of Papua New Guinea National Department of Health, Port Moresby, Papua New Guinea; 3 World Health Organization Representative Office for Papua New Guinea, Port Moresby, Papua New Guinea

**Keywords:** Systematic review, Infections, diseases, disorders, injuries, Control strategies

## Abstract

**Background:**

Yaws is a chronic infectious disease that disproportionately affects under-reached communities. In 2023, Papua New Guinea (PNG) reported 100 165 suspected yaws cases, accounting for 45% of cases reported worldwide. The WHO targets yaws eradication by 2030, primarily through mass drug administration (MDA) of azithromycin. This study evaluates the impact of an integrated MDA programme in PNG and reviews global evidence on optimal MDA strategies through a network meta-analysis (NMA).

**Methods:**

A comparative observational study was undertaken analysing outpatient department (OPD) attendances between West New Britain province (WNB; intervention) and New Ireland province (NIP; comparator) from November 2023 to August 2024. The intervention in WNB involved a single-round, integrated four-drug MDA provided in December 2023, comprising azithromycin, ivermectin, diethylcarbamazine and albendazole. Temporal trends in OPD attendances were analysed using negative binomial regression. A Bayesian NMA of six studies identified through a systematic review compared the effectiveness of azithromycin-based regimens for yaws eradication.

**Results:**

Between Q3 2023 and Q1 2024, yaws-related OPD attendances in WNB declined significantly, with a 41% (p<0.0001) reduction relative to the comparator province (NIP). This reduction was sustained for at least 6 months following MDA. Over the same period, a 33% decline in total skin-related outpatient attendances was observed. In the NMA, three rounds of azithromycin MDA were associated with lower odds of active yaws at follow-up than observation (OR 0.04, 95% CrI 0.005 to 0.24) and had the highest probability of being ranked most effective. Single-round azithromycin MDA was also associated with reduced odds of active yaws at follow-up (OR 0.15, 95% CrI 0.05 to 0.45).

**Conclusion:**

In a high-burden setting, a single round of integrated MDA was associated with substantial early reductions in yaws burden. Multi-round azithromycin strategies, particularly three-round regimens, were associated with larger and more sustained reductions in community prevalence. These findings inform considerations for yaws control efforts in settings with persistent transmission.

**WHAT IS ALREADY KNOWN ON THIS TOPIC**

- Yaws is targeted for global eradication by 2030, and Papua New Guinea (PNG) reported 100 165 notified yaws cases in 2023, representing the highest national caseload globally.
- Oral azithromycin mass drug administration (MDA) has been shown to rapidly reduce active yaws prevalence at the community level, although sustained interruption of transmission has proven challenging in some high-prevalence and high-transmission settings.

**WHAT THIS STUDY ADDS**

- This study provides a comparative evaluation of an integrated four-drug MDA campaign in PNG, demonstrating a 41% reduction in reported yaws cases and a 33% reduction in total skin-related outpatient attendance following implementation.
- A network meta-analysis indicates that a three-round azithromycin regimen achieves greater and more sustained reductions in yaws prevalence than the standard single-round regimen across available comparative evidence.

**HOW THIS STUDY MIGHT AFFECT RESEARCH, PRACTICE OR POLICY**

- The findings suggest that intensified, multi-round MDA schedules may be required in hyper-endemic settings to achieve sustained reductions in transmission, particularly where latent infection reservoirs are large.
- The operational experience of the integrated campaign supports consideration of integrated skin-NTD delivery platforms within broader national and subnational strategies, in line with current priorities on programme integration.

## Introduction

 Yaws, a chronic infectious disease caused by the bacterium *Treponema pallidum* subsp. *pertenue*, remains a significant public health burden in tropical areas. It disproportionately affects children under 15 years of age and under-reached communities with limited access to healthcare and sanitation.^1 2^ Globally, the impact of yaws is substantial; without sustained eradication efforts, an estimated 1.6 million disability-adjusted life-years (DALYs) are projected to be lost between 2015 and 2050.^3^ This figure, primarily capturing health loss from disability, likely underestimates the true societal impact, as yaws also inflicts considerable stigma, social exclusion and educational disruption, which are not fully encapsulated by DALY calculations.^4^

The Western Pacific Region bears the highest known burden of yaws globally. Papua New Guinea (PNG) reported 96 000 suspected yaws cases in 2022 and 100 165 suspected cases in 2023, which accounted for approximately 45% of all cases reported worldwide in that year.^2 5^ The WHO has prioritised the eradication of yaws by 2030.^6^ Current global efforts to interrupt yaws transmission have focused on oral azithromycin mass drug administration (MDA), following strategies introduced during the past decade.^7 8^ These strategies typically involve an initial round of total community treatment (TCT), in which all members of an affected community are offered treatment, followed by targeted treatment approaches focusing on active cases and their contacts, with the aim of interrupting transmission in endemic populations.^9^

The first part of the study evaluates the impact of PNG’s first pivotal four-drug (azithromycin, ivermectin, diethylcarbamazine and albendazole) integrated single-round MDA campaign in West New Britain Province (WNB), implemented in December 2023, by analysing the monthly trend of outpatient department (OPD) attendances. By comparing outcomes with New Ireland Province (NIP), a province with a similar yaws burden that has not undergone MDA, the results provide actionable insights into the determinants of successful yaws eradication.

In the second part of the study, a systematic review of azithromycin treatment regimens in yaws eradication was conducted. While multiple clinical trials have evaluated the effectiveness of azithromycin-based MDA regimens, direct head-to-head comparisons between all potential strategies in yaws eradication remain limited. Network meta-analysis (NMA) enables the simultaneous comparison of the effectiveness of multiple interventions by synthesising both direct and indirect evidence from existing studies.^10 11^ This dual approach aims not only to identify which regimens are most effective but also to illuminate how these strategies can be adapted and implemented in high-burden settings.

## Methods

### Observational study

This study evaluated the first round of MDA in WNB, PNG, which took place from late November 2023 to January 2024, with a subsequent mop-up phase in March 2024. NIP, which did not implement MDA during the study period, was included as a contemporaneous comparison. The campaign targeted the total provincial population (n=320 071) across the three districts of Talasea, Kandrian-Gloucester and Nakanai. The intervention utilised an integrated four‐drug regimen comprising ivermectin, diethylcarbamazine and albendazole (IDA) co-administered with azithromycin.

Drug delivery utilised a multi-channel strategy to maximise coverage and equity. School-aged children were treated in educational settings, while the broader community was reached through fixed distribution points at 37 health facilities and designated community booths. These were supplemented by workplace clinics and mobile teams providing direct house-to-house, observed treatment. Standard exclusion criteria, including pregnancy and age restrictions, were applied. Province-wide coverage reached 63.1% for the IDA regimen and 54.8% for azithromycin, as documented in electronic health registries and confirmed by a post-MDA coverage evaluation survey.

Community engagement was an integral component of implementation. Provincial health teams conducted consultations with village chiefs, church leaders and women’s groups to explain the intervention and address safety concerns. Feedback from these sessions informed logistical decisions, including the prioritisation of community-based distribution points, and village health volunteers supported social mobilisation and community communication.

OPD attendances were analysed as anonymised monthly counts and summarised by quarter for reporting. Yaws diagnoses in the routine outpatient dataset were based primarily on clinical assessment, with serological confirmation where available. Trends in yaws-related OPD attendances between WNB (intervention) and NIP (comparison) were analysed using a negative binomial generalised linear model to account for overdispersion in the count data. The model included fixed effects for province and time and a province-time interaction term to estimate the treatment effect. Statistical significance was defined as p<0.05.

### Systematic review and network meta-analysis

A systematic review and NMA were conducted in accordance with PRISMA and PRISMA-NMA guidelines.^10 12^ A protocol was developed a priori but was not registered. The protocol was finalised prior to study screening and data extraction and was not amended during the review process. PubMed, MEDLINE, Embase and ClinicalTrials.gov were searched from 2000 to December 2024 for randomised controlled trials, cluster randomised trials and observational studies conducted in yaws-endemic populations.

Eligible interventions comprised azithromycin-based regimens (single-round or multi-round) and benzathine benzylpenicillin, compared with active surveillance or alternative antibiotic strategies. Primary outcomes were reductions in yaws prevalence and cure or serological response rates. Animal studies, modelling studies, non-therapeutic interventions and studies without comparative outcome data were excluded. Searches were limited to English-language publications. The full search strategy is provided in the online supplemental appendix 1. Two reviewers independently screened records and extracted data, with disagreements resolved by consensus. Risk of bias was assessed independently by two reviewers using the RoB 2 (Cochrane Risk of Bias tool 2) for randomised trials and the ROBINS-I (Risk Of Bias In Non-randomised Studies - of Interventions) tool for non-randomised studies.^13 14^ An NMA was performed using BUGSnet (v1.1.2) and rjags (v4-16) in R (v4.4.2) to estimate odds ratios (ORs) with 95% credible intervals (CrI).^15^ The NMA included comparative studies of azithromycin-based regimens for yaws delivered in different programmatic settings and schedules. Outcomes were analysed at the individual level, and effect estimates represent OR for active yaws measured at study-specific follow-up time points. In studies reporting cure, cured participants were classified as not having active yaws at follow-up. Analyses were performed within a Bayesian framework using Markov chain Monte Carlo simulation. A random-effects model with default prior heterogeneity parameters was selected over a fixed-effects model based on a lower Deviance Information Criterion (DIC). Four chains with different initial values were initiated simultaneously, with 1000 adaptations, 10 000 burn-ins and 50 000 iterations. Model convergence was assessed using Gelman–Rubin–Brooks diagnostics in addition to trace and density plots.^16^ The assumption of transitivity was assessed by comparing study populations, interventions, outcomes and follow-up durations across comparisons, while consistency between direct and indirect evidence was evaluated using a global inconsistency model.^17^ Treatments were ranked using the Surface Under the Cumulative Ranking Curve (SUCRA), where higher values indicate a greater probability of a treatment being among the most effective options within the network.^11^ Certainty of evidence was assessed qualitatively, taking into account study design, risk of bias, imprecision and consistency across the network; a formal GRADE assessment was not undertaken due to network sparsity and the inclusion of non-randomised evidence for some comparisons.^18^ Assessment of publication bias and small-study effects was not performed because the number of studies contributing to each comparison was insufficient to support reliable evaluation.

#### Patient and public involvement

Patients and the public were not involved in the design, conduct, reporting or dissemination plans of either the observational study or the systematic review. This research consisted of the analysis of anonymised, routine national programme data and a review of existing literature. Although the implementation of the MDA in WNB involved extensive community engagement to inform logistical delivery, these stakeholders were not involved in the formal development of the research questions, the statistical analysis or the drafting of this manuscript.

## Results

### Impact of integrated MDA in West New Britain

**Figure 1:**
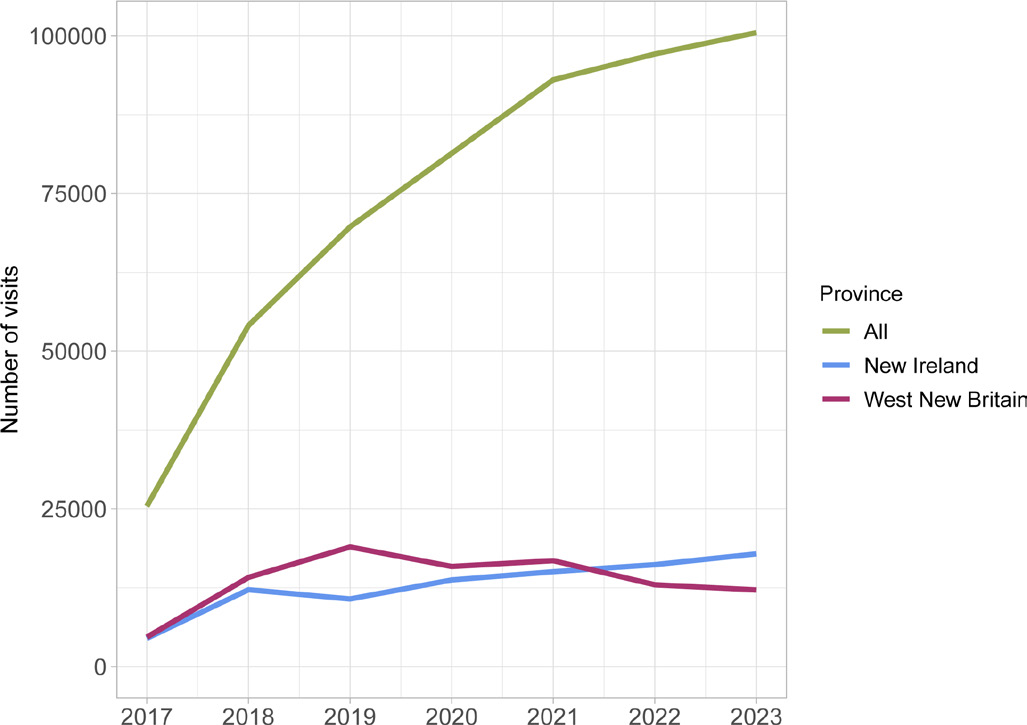
Number of visits for yaws in OPD in all provinces, WNB and NIP from 2017 to 2023. OPD, outpatient department; NIP, New Ireland province; WNB, West New Britain province.

Prior to the intervention, PNG experienced a substantial year-on-year increase in yaws-related outpatient presentations. The total number of OPD visits for yaws across all provinces rose steeply from 25 444 in 2017 to over 100 000 in 2023 (figure 1). This represents an almost fourfold increase over the 6-year period, contributing to a reported fivefold increase overall between 2013 and 2023. Analysis of provincial trends indicated that WNB and NIP shared broadly similar baseline numbers of yaws visits prior to the intervention period.

Following the implementation of the integrated four-drug MDA in WNB in late 2023, a significant reduction in OPD attendances for all skin diseases was observed. Comparing the pre-MDA baseline (Q3 2023) to the post-MDA period (Q1 2024), WNB recorded a 33% reduction in overall OPD attendances for skin diseases (p<0.0001) (online supplemental figure). In contrast, NIP, which did not undergo MDA, showed no significant change, recording only a 2% reduction in overall skin disease attendances over the same period.

**Figure 2:**
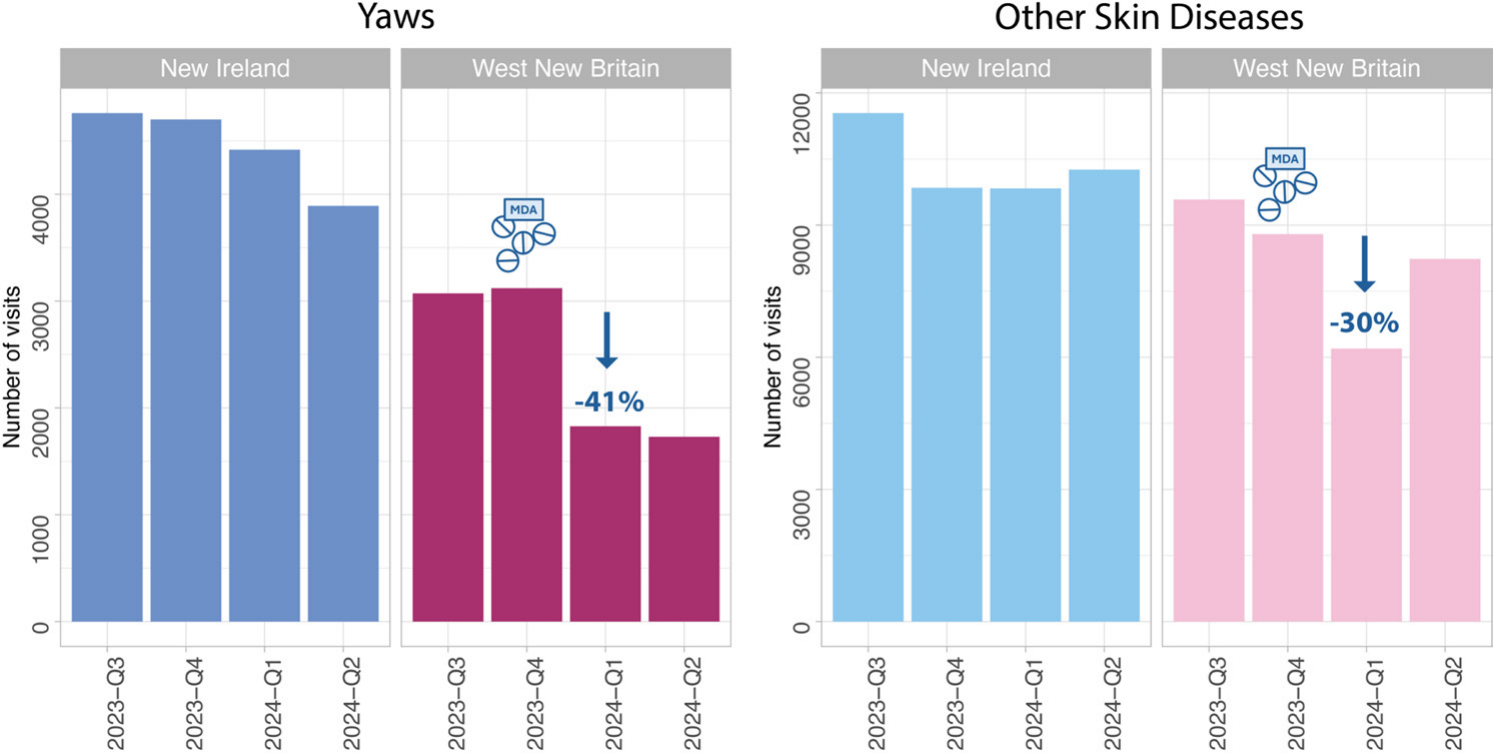
OPD visits for yaws and other skin diseases in WNB (dark pink/light pink) show a marked decrease from Q3 2023 to Q1 2024, while visits in NIP (dark blue/light blue) fluctuated without significant trends. MDA, mass drug administration; OPD, outpatient department; NIP, New Ireland province; WNB, West New Britain province.

Operational surveillance reports indicate that yaws cases reported in OPDs decreased by 48% in 6 months following the MDA compared with the pre-MDA baseline. Consistent with this, the negative binomial model estimated a 41% adjusted decline in yaws-related outpatient attendances in WNB relative to NIP between Q3 2023 and Q1 2024 (p<0.0001) (figure 2), with this reduction maintained through Q2 2024. During the same period, attendances for other (non-yaws) skin diseases in WNB also declined by 30%.

In the comparator province (NIP), yaws OPD visits fluctuated without a consistent downward trend over the corresponding period. The decline in overall OPD attendances observed in WNB during the first half of 2024 (Q1–Q2) was significantly greater than that in NIP (p<0.0001), widening the gap in disease burden between the two provinces post-intervention. The most substantial divergence occurred in Q1 2024 (p<0.0001). Crucially, yaws OPD attendance in WNB remained at this reduced level for at least 6 months post-MDA (through Q2 2024). While a slight increase in attendances for other skin diseases was observed in WNB by the 6-month follow-up (Q2 2024), yaws attendances continued to be markedly lower than pre-MDA levels.

### Systematic review and study characteristics

**Figure 3:**
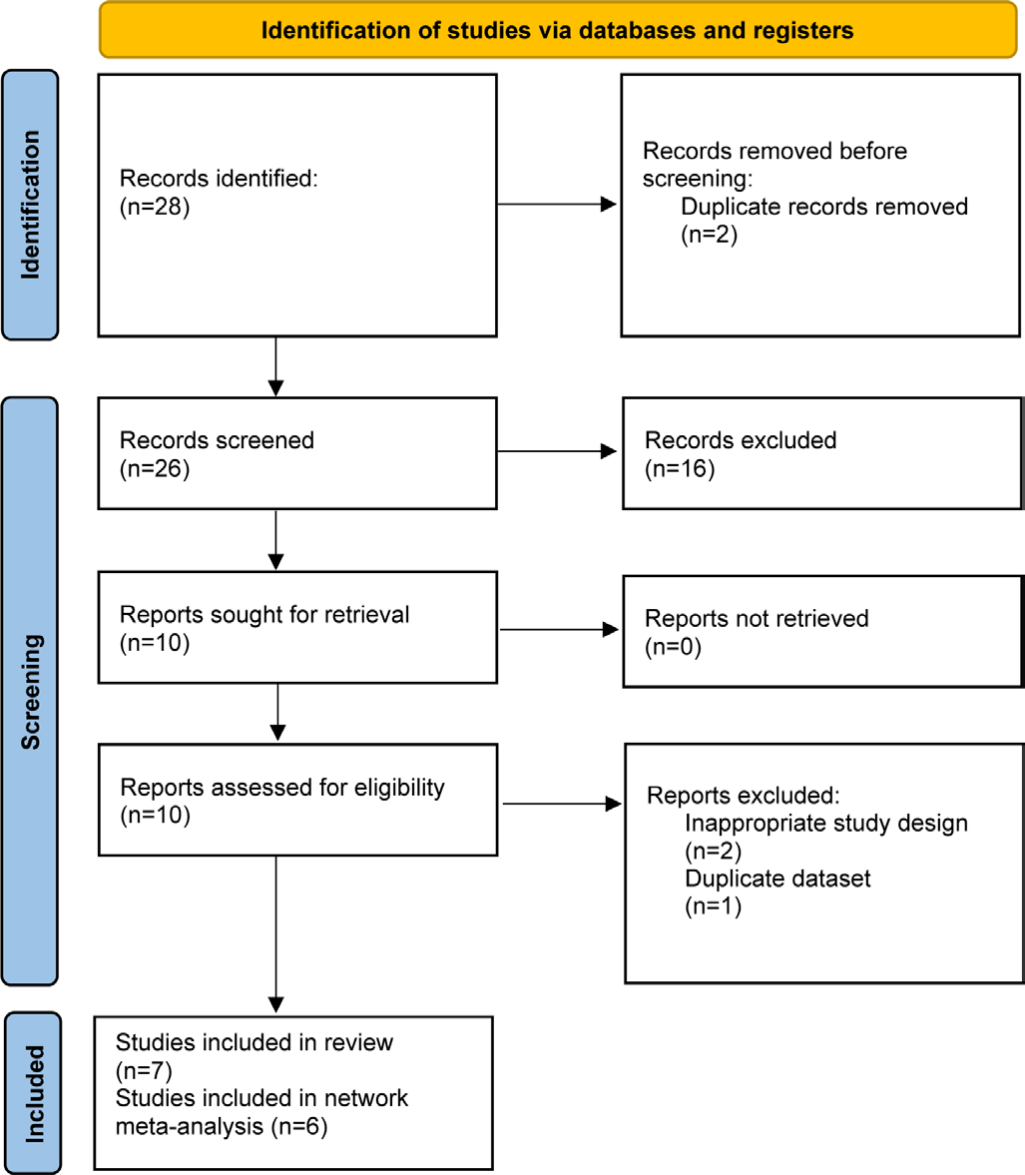
PRISMA 2020 flow diagram of study selection. A flow diagram showing identification, screening, eligibility assessment and inclusion of studies for the systematic review and network meta-analysis, based on searches of bibliographic databases and trial registers only. PRISMA, Preferred Reporting Items for Systematic Reviews and Meta-Analyses.

The systematic review identified seven eligible studies (figure 3). The review included 90 552 participants from studies conducted in Ghana and PNG, representing yaws-endemic settings in West Africa and the Western Pacific (table 1). Study designs comprised three individually randomised controlled trials, one cluster-randomised trial and three observational studies.

**Table 1:** Characteristics and key findings of studies included in the systematic review

Study	Design	Country (site)	Participants/population	Intervention	Follow-up	Main finding
Mitjà *et al*^29^	Open-label non-inferiority RCT	PNG (Lihir)	Children aged 6 months to 15 years with serologically confirmed yaws (n=250)	Oral azithromycin 30 mg/kg vs intramuscular benzathine benzylpenicillin	6 months	Cure at 6 months: 96% vs 93%; risk difference −3.4% (95% CI −9.3 to 2.4).
Kwakye-Maclean *et al*^30^	Open-label non-inferiority RCT	Ghana	Children aged 1 to 15 years with serologically confirmed yaws (n=353)	Oral azithromycin 30 mg/kg vs intramuscular benzathine penicillin	6 months	Serological cure at 6 months: 57.4% vs 49.1%.
Marks *et al*^31^	Open-label non-inferiority RCT	Ghana and PNG	Children aged 6 to 15 years with serologically confirmed yaws (n=583)	Oral azithromycin 30 mg/kg vs 20 mg/kg	6 months	Cure at 6 months: 84.0% vs 80.3%; difference 3.7% (95% CI −8.4 to 15.7).
John *et al*^22^	Open-label cluster-randomised trial	PNG (Namatanai)	Community residents in 38 wards (n=56 676)	One round of TCT with oral azithromycin 30 mg/kg plus targeted treatment vs three rounds of TCT with oral azithromycin 30 mg/kg at 6-month intervals	6 months after final round (*18 months from baseline)	Active yaws prevalence at 18 months: 0.16% vs 0.04%; adjusted RR 4.08 (95% CI 1.90 to 8.76).
Mitjà *et al*^32^	Community intervention study	PNG (Lihir)	Community residents (n=16 092)	One round of total community treatment with oral azithromycin 30 mg/kg	6 and 12 months	Active yaws prevalence decreased from 2.4% at baseline to 0.3% at 6 months and remained 0.3% at 12 months.
Abdulai *et al*^33^	Community intervention pilot study	Ghana (Abamkrom)	Community residents (n=16 287)	One round of total community treatment with oral azithromycin 30 mg/kg	12 months	Active yaws from 5.7% to 0.6% at 12 months.
Mitjà *et al*^19^	Cohort study	PNG (Lihir)	Children aged under 20 years with serologically confirmed yaws (n=311)	Single-dose oral azithromycin 30 mg/kg	6, 12 and 24 months	Serological cure at 6, 12 and 24 months was 88%, 90% and 92% in latent yaws and 83%, 89% and 94% in active yaws.

CI, confidence Interval; RCT, randomised controlled trial; RR, risk ratio; TCT, total community treatment.

The quality of evidence varied by study design (online supplemental appendix 2). Randomised controlled trials were judged to have a low risk of bias, reflecting appropriate randomisation and outcome assessment. Observational studies were judged to have a serious risk of bias, driven primarily by confounding inherent to non-randomised designs. These differences were considered in the interpretation of the findings.

### Network meta-analysis

**Figure 4:**
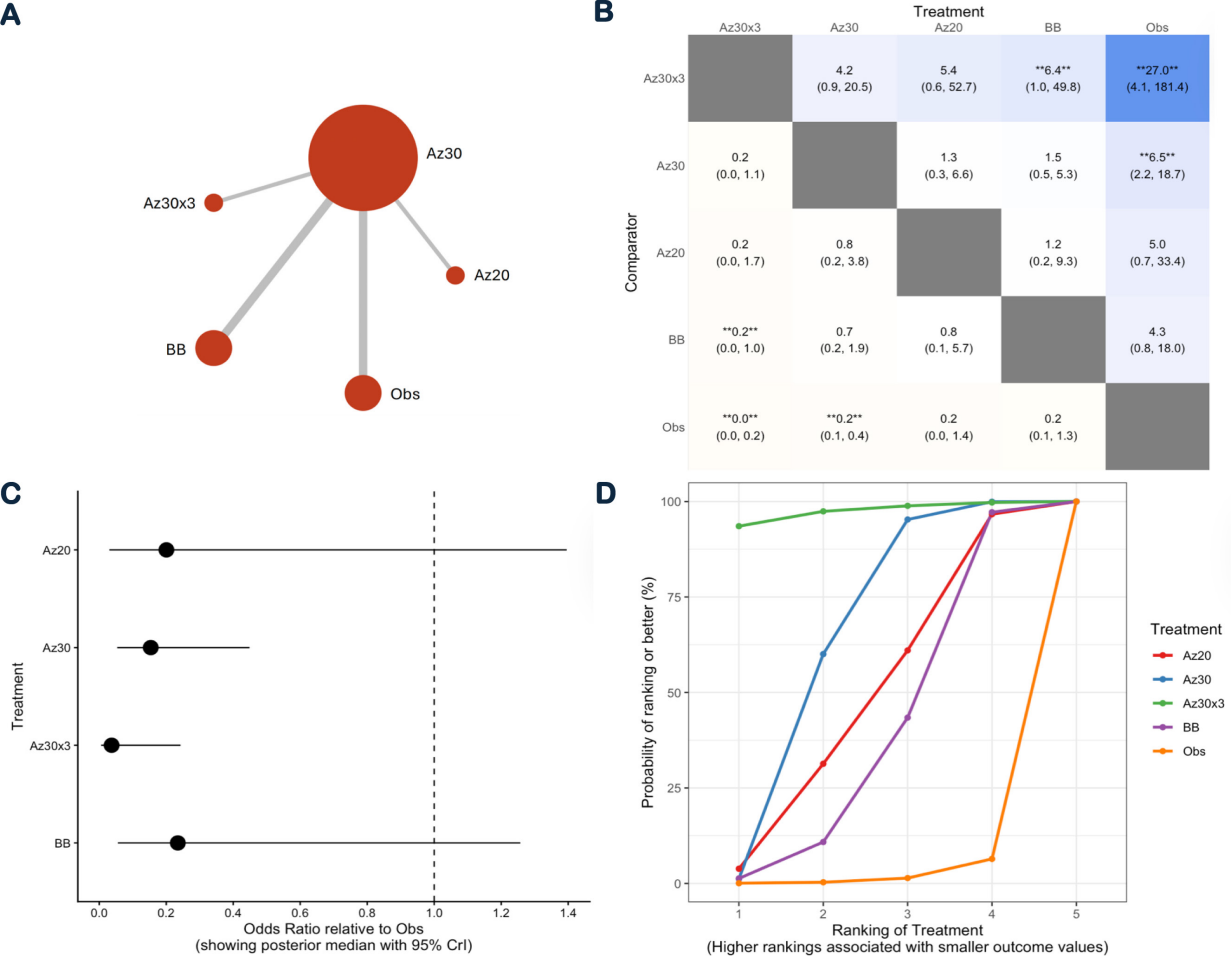
Network meta-analysis of treatments for yaws. (**A**) Network plot including four interventions and observation: single-round Az30, single-round Az20, three rounds of Az30×3, BB and Obs. Node size is proportional to the number of participants and edge thickness to the amount of direct evidence. (**B**) League table showing pairwise comparisons among Az30, Az20, Az30×3, BB and Obs, reported as ORs with 95% CrI. (**C**) Forest plot of posterior median ORs with 95% CrI for each active intervention (Az30, Az20, Az30×3 and BB) compared with observation (Obs). (**D**) Rankograms showing the posterior probability of each intervention being ranked from most to least effective within the Bayesian network meta-analysis. Az20, azithromycin 20 mg/kg; Az30, azithromycin 30 mg/kg; Az30×3, three-round azithromycin 30 mg/kg; BB, benzathine benzylpenicillin; Obs, observation.

Six studies comprising 90 241 participants contributed to the NMA. Mitjà et al.(2017) was excluded from the NMA because it did not provide a between-intervention comparison.^19^ The NMA compared four regimens with observation, using individual-level active yaws outcomes measured at study-specific follow-up time points (figure 4A). Single-round azithromycin 30 mg/kg (Az30) formed the central node of the network, linking benzathine benzylpenicillin (BB) and the intensified three-round azithromycin strategy (Az30×3), as well as low-dose azithromycin (Az20).

Relative treatment effects are presented in the league table (figure 4B) and forest plot (figure 4C). ORs are reported as the odds of yaws relative to observation, with values below 1 indicating a beneficial effect. Compared with observation, the Az30×3 regimen was associated with substantially lower odds of active yaws (OR 0.04, 95% CrI 0.005 to 0.24). Single-round Az30 was also associated with reduced odds of active yaws relative to observation (OR 0.15, 95% CrI 0.05 to 0.45), although it was less effective than the three-round regimen in indirect comparisons. Low-dose Az20 was associated with an OR of 0.20 (95% CrI 0.03 to 1.40), while benzathine benzylpenicillin demonstrated an OR of 0.23 (95% CrI 0.06 to 1.26). For both interventions, credible intervals crossed the null value, indicating substantial uncertainty in effect estimates. The wide credible intervals observed across several comparisons reflect network sparsity and limited direct evidence for some treatment nodes.

Sensitivity analyses assessing follow-up duration and intervention approach, using meta-regression models with a binary covariate for community-wide total community treatment, did not materially alter the findings or improve model fit. Consistency between direct and indirect evidence was assessed using a global inconsistency model, with no evidence of important inconsistency identified within the network. Assessment of small-study effects was not undertaken due to the limited number of studies contributing to each comparison.

The Surface Under the Cumulative Ranking Curve (SUCRA) analysis is shown in figure 4D. Az30×3 had the highest probability of being the most effective intervention, followed by single-round Az30. Treatment rankings should be interpreted in light of uncertainty in effect estimates and network sparsity.

## Discussion

This study integrates evidence from an NMA and a large observational evaluation to examine MDA strategies for yaws control and eradication. Across both analytical approaches, the findings indicate that a single round of total community treatment (TCT), followed by targeted treatment, may be insufficient to achieve sustained interruption of transmission in hyper-endemic settings. While single-round strategies consistently produce marked short-term reductions in active disease, evidence from longitudinal follow-up and comparative trials suggests that these effects are often not maintained over time.^20 21^

Within the NMA, the three-round azithromycin regimen demonstrated the largest estimated effect relative to active surveillance and the highest probability of being ranked most effective. Although effect estimates were characterised by wide credible intervals, the direction and relative magnitude of effects are consistent with the only large cluster-randomised trial to directly compare intensified and standard strategies, which reported significantly lower community prevalence following three rounds of mass azithromycin delivered at 6-month intervals.^22^ In contrast, the standard single-round azithromycin regimen, which has been widely implemented in yaws control programmes, showed smaller and less certain effects.

The observational evaluation from WNB provides complementary programme-level evidence. The integrated MDA campaign was associated with substantial reductions in reported yaws cases and OPD attendances, indicating a meaningful immediate impact on disease burden. However, experience from previous campaigns indicates that reductions in clinical disease do not necessarily equate to sustained interruption of transmission, particularly in settings characterised by high baseline prevalence, intense transmission pressure and limited post-MDA surveillance.^19 21^ These findings underscore the distinction between short-term control and sustained reduction.

### Interpretation and implications for programmes and integration

A central challenge for yaws eradication is the reservoir of latent infection, which is not detectable through routine clinical surveillance. Individuals with latent yaws substantially outnumber those with active disease and can sustain transmission even when clinical incidence declines.^21^ In this context, single-round strategies may be vulnerable to reduced impact if coverage is incomplete or unevenly distributed.

Coverage heterogeneity further undermines the effectiveness of one-off interventions. Operational data from WNB illustrate differential uptake across drugs within an integrated campaign, with lower coverage for azithromycin than for the lymphatic filariasis regimen. Such heterogeneity is well recognised in integrated delivery platforms, where differences in perceived relevance, risk perception and messaging can influence participation.^23^ From a transmission perspective, even modest gaps in coverage may allow rapid recrudescence when a substantial latent reservoir persists.

Reported azithromycin coverage reflects uptake across the total eligible population and may therefore underestimate coverage in children, who represent the primary reservoir of transmission.^7^ Differential uptake across age groups, particularly through school-based delivery, may partly contribute to the observed reductions despite moderate overall coverage. In addition, yaws transmission is spatially clustered, and treatment of core transmission groups may result in disproportionate reductions in observed disease.^21^

The apparent advantage of multi-round strategies is therefore both biologically and operationally plausible. Repeated rounds increase the likelihood of reaching individuals missed previously, treating infections acquired after earlier distributions and progressively depleting the latent reservoir. These findings align with modelling work suggesting that in high-transmission settings, single-round strategies are unlikely to reduce transmission below the replacement threshold unless coverage is exceptionally high and sustained.^24^ These findings should also be interpreted in the context of reported macrolide resistance in *Treponema pallidum* subsp. *pertenue* in PNG, highlighting the importance of surveillance for treatment failure and resistance as repeated azithromycin-based strategies are considered.^20^,^22^

Importantly, the findings also align with the strategic direction articulated in the Neglected Tropical Diseases Roadmap 2021–2030, which emphasises the need to accelerate programmatic action, tailor interventions to epidemiological context and move beyond uniform approaches in settings with persistent transmission.^6^ The road map highlights intensified disease management and integration across NTDs as key enablers of elimination and eradication goals.

Integration with skin-NTD, communicable diseases or routine health programmes may therefore be critical to the feasibility and sustainability of intensified yaws strategies. Yaws shares epidemiological and operational overlap with other skin-related NTDs, including scabies and leprosy, and integrated approaches have been shown to improve efficiency, community engagement and case detection.^25 26^ Embedding yaws surveillance, follow-up and response within primary healthcare systems may further support country ownership and reduce reliance on repeated vertical campaigns, consistent with the NTD roadmap principles.^27^ This approach is consistent with the 2025 WHO–UN agreement on integrated action for skin-related neglected tropical diseases, which calls for coordinated delivery platforms, shared surveillance systems and stronger integration with primary healthcare.^28^

### Strengths and limitations

This study integrates comparative evidence synthesis with evaluation of a large-scale implementation programme, providing complementary insights into intervention effectiveness and delivery in routine practice. The NMA enables structured comparison of multiple treatment strategies where direct head-to-head evidence is limited, while the observational analysis contextualises these findings within standard programme delivery. Inclusion of a contemporaneous comparison province strengthened inference for the observational analysis.

Several limitations warrant consideration. Certainty of evidence was assessed qualitatively rather than using a formal GRADE approach, and treatment rankings should therefore be interpreted with caution. Evidence informing intensified regimens remains limited, with wide credible intervals reflecting imprecision and network sparsity. Some estimates were derived from non-randomised studies, introducing the potential for residual confounding despite formal risk of bias assessment. Formal assessment of publication bias and small-study effects was not feasible because the number of studies contributing to each comparison was insufficient to support reliable evaluation. Although no important inconsistency between direct and indirect evidence was identified, the validity of network estimates depends on assumptions of transitivity, including similarity of study populations, interventions and outcome definitions across studies.

The observational analysis relied on routine health information system data, which may be influenced by changes in health-seeking behaviour, diagnostic practices and reporting completeness following MDA. The increase in reported yaws presentations may reflect a combination of changes in case detection and reporting within healthcare services and a continuing underlying burden of disease. The true burden is difficult to quantify without systematic mapping and serology-based testing. Yaws diagnoses in the routine outpatient dataset were based primarily on clinical assessment, and misclassification with other causes of ulcerative skin disease is possible. Routine surveillance data remain useful for assessing programme-level changes in reported yaws burden under routine service conditions. Although inclusion of a contemporaneous comparator province strengthens inference, unmeasured contextual differences cannot be excluded.

## Conclusion

In conclusion, evidence from both network synthesis and observational implementation suggests that single-round MDA strategies may be insufficient to achieve sustained interruption of yaws transmission in hyper-endemic settings. Multi-round azithromycin regimens appear more effective in reducing community prevalence over extended periods, noting that the available evidence is subject to uncertainty and limited direct comparative data. Progress towards yaws eradication will depend on strategies that address latent infection reservoirs, coverage heterogeneity and operational realities and that are delivered through integrated platforms aligned with the NTD road map’s emphasis on intensified action and integration.

## Supplementary material

10.1136/bmjgh-2026-023743 online supplemental figure 1

10.1136/bmjgh-2026-023743 online supplemental appendix 1

10.1136/bmjgh-2026-023743 online supplemental appendix 2

## Data Availability

Data may be obtained from a third party and are not publicly available.
