# Supplementary figures and images for: Integrated mass drug administration for yaws eradication: evidence from a comparative observational study in Papua New Guinea and a systematic review with network meta-analysis
Source: BMJ Glob Health. 2026 May 13;11(5):e023743. doi: 10.1136/bmjgh-2026-023743 (PMC13182430; doi:10.1136/bmjgh-2026-023743)

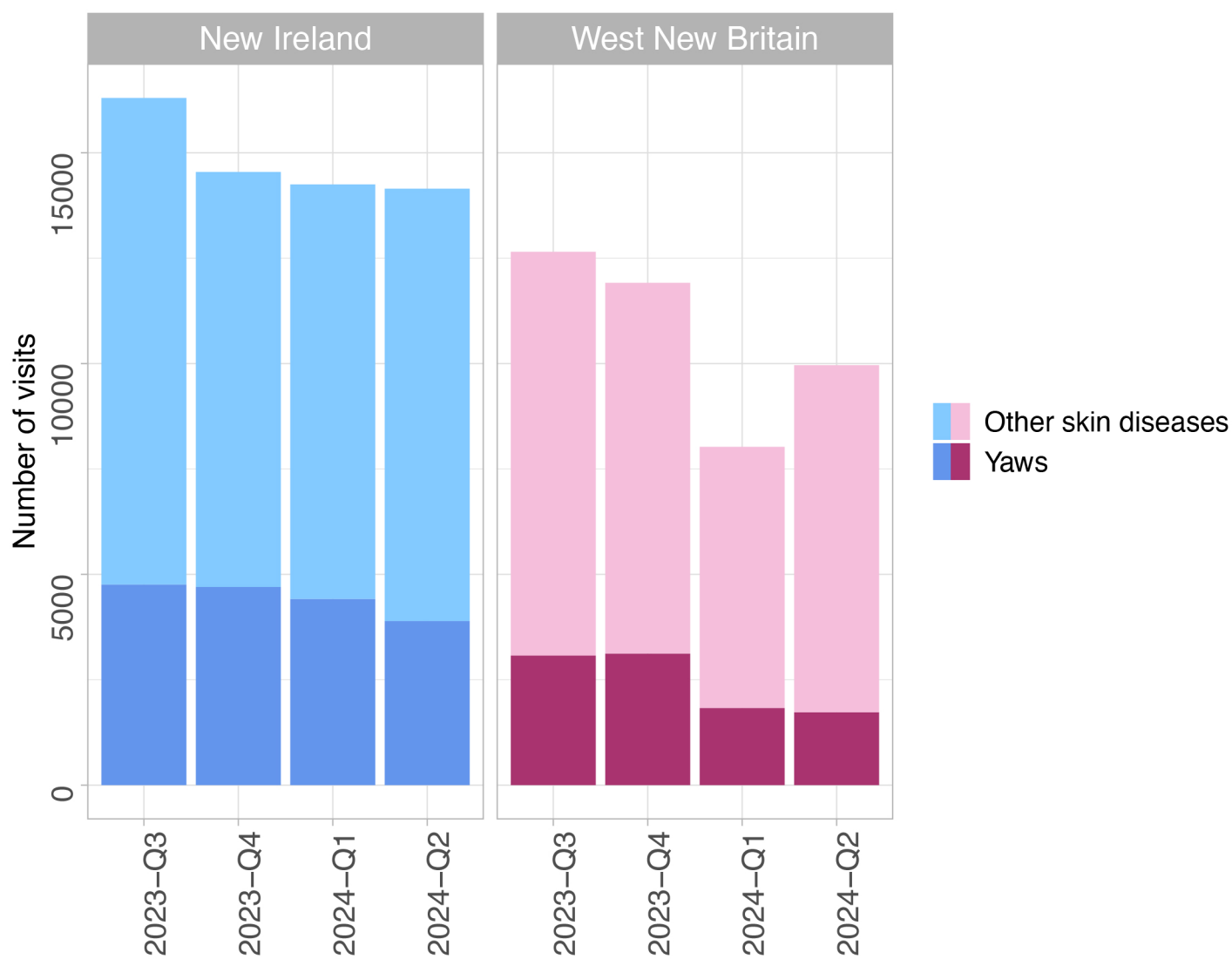

Supplement: online supplemental figure 1 [file bmjgh-11-5-s001.pdf]
