# Supplementary material for: Integrated mass drug administration for yaws eradication: evidence from a comparative observational study in Papua New Guinea and a systematic review with network meta-analysis
Source: BMJ Glob Health. 2026 May 13;11(5):e023743. doi: 10.1136/bmjgh-2026-023743 (PMC13182430; doi:10.1136/bmjgh-2026-023743)
Supplement: online supplemental appendix 1 [file bmjgh-11-5-s002.docx]

# **Supplementary Appendix I. Search strategy**

**Databases**
MEDLINE (Ovid), Embase (Ovid), PubMed, and ClinicalTrials.gov were searched from inception to December 2024. Searches in MEDLINE and Embase were limited to English-language publications.

## MEDLINE (Ovid)

| Line | Search terms |
| --- | --- |
| 1 | Yaws/ OR frambesia.mp. OR Treponema pallidum pertenue.mp. |
| 2 | Neglected Tropical Diseases/ |
| 3 | 1 OR 2 |
| 4 | Mass Drug Administration.mp. OR mass treatment.mp. OR total community treatment.mp. |
| 5 | Azithromycin/ OR azithromycin.mp. |
| 6 | Penicillin G Benzathine/ OR benzathine penicillin.mp. |
| 7 | 4 OR 5 OR 6 |
| 8 | Clinical Trial.pt. OR Randomized Controlled Trial.pt. |
| 9 | 8 |
| 10 | 3 AND 7 AND 9 |

Limits: 2000–2024; English.

## Embase (Ovid)

The MEDLINE strategy was translated using Emtree terms and Embase syntax for yaws, azithromycin, benzathine penicillin, and mass drug administration. Limits: 2000–2024; English.

## PubMed

(yaws OR frambesia) AND (azithromycin OR "mass drug administration" OR "mass treatment")

## ClinicalTrials.gov

Search terms: yaws; azithromycin; mass drug administration.

## Study selection

Records were deduplicated and screened independently by two reviewers. The selection process is shown in the PRISMA flow diagram.
