# Supplementary material for: Integrated mass drug administration for yaws eradication: evidence from a comparative observational study in Papua New Guinea and a systematic review with network meta-analysis
Source: BMJ Glob Health. 2026 May 13;11(5):e023743. doi: 10.1136/bmjgh-2026-023743 (PMC13182430; doi:10.1136/bmjgh-2026-023743)
Supplement: online supplemental appendix 2 [file bmjgh-11-5-s003.docx]

### **Supplementary Appendix II.**

### **Risk of bias assessment**

**Table 1a. Risk of bias (RoB 2) assessment for randomised controlled trials**

| **Study** | **Randomisation process** | **Deviations from intended interventions** | **Missing outcome data** | **Measurement of the outcome** | **Selection of the reported result** | **Overall risk of bias** |
| --- | --- | --- | --- | --- | --- | --- |
| **Mitjà et al. (2012)** | Low | Low | Low | Low | Low | Low |
| **Kwakye-Maclean et al. (2017)** | Low | Low | Low | Low | Low | Low |
| **Marks et al. (2018)** | Low | Some concerns | Some concerns | Some concerns | Low | Some concerns |
| **John et al. (2022)** | Low | Low | Low | Low | Low | Low |

**Table 1b. Risk of bias (ROBINS-I) assessment for non-randomised studies**

| **Study** | **Confounding** | **Selection of participants** | **Classification of interventions** | **Deviations from intended interventions** | **Missing data** | **Measurement of outcomes** | **Selection of reported result** | **Overall risk of bias** |
| --- | --- | --- | --- | --- | --- | --- | --- | --- |
| **Mitjà et al. (2015)** | Serious | Low | Low | Low | Low | Low | Low | Serious |
| **Abdulai et al. (2018)** | Serious | Serious | Low | Low | Moderate | Moderate | Low | Serious |
| **Mitjà et al. (2017)** | Moderate | Moderate | Low | Low | Low | Low | Low | Moderate |
